# Agronomic, Genetic and Quantitative Trait Characterization of Nightshade Accessions

**DOI:** 10.3390/plants11111489

**Published:** 2022-05-31

**Authors:** Ntombifuthi Msewu Mabuza, Sydney Mavengahama, Motlogeloa Mokolobate

**Affiliations:** Food Security and Safety Focus Area and School of Agricultural Sciences, Faculty of Natural and Agricultural Sciences, North-West University, Mmabatho 2745, South Africa; sydney.mavengahama@nwu.ac.za (S.M.); motlogeloa.mokolobate@nwu.ac.za (M.M.)

**Keywords:** correlation, genetic advance, heritability, morphological, solanum, PCA: variability

## Abstract

Nightshades are among many underutilized and neglected African indigenous leafy vegetable (AILVs) species, and if adequately exploited, they could improve food, nutrition and income among the rural population. Morphological characterization of available accessions is key for the breeder to identify and select superior accessions as parents for utilization in breeding programs. Fifteen accessions of nightshade were evaluated for morpho-agronomic variation in an open field trial implemented in a randomized complete block design with three replicates across the two growing seasons. The accessions exhibited significant (*p* < 0.0001) differences in all quantitative traits. The data analysis showed that Scabrum (805.30 g/plant) followed by Ncampus (718.60 g/plant) produced the highest fresh leaf yield; for fruit fresh yield, the accession NigSN18 (1782.00 g/plant) recorded the highest, followed by ManTown (1507.90 g/plant). The accession N5547 had the tallest plants (66.83 cm), followed by accession Timbali (62.31 cm). The first four principal components (PCs) accounted for 86.82% of the total variation, which had an eigenvalue greater than 1. The cluster analysis grouped the accessions into 14 clusters based on their genetic similarity. Results of genetic studies revealed that phenotypic coefficient variation was higher than genotypic coefficient of variation for all parameters evaluated, indicating the environmental influence on the expression of these traits. Both GCV and PCV were higher for the largest leaf area, moderate to high for the remaining characters and low for leaf fresh yield per plant. High heritability coupled with genetic advance as a mean percentage (H^2^-70.59%, GAM-142.4%), indicating the presence of additive gene effects. Hence, selection can be employed for the improvement of this trait in nightshades. The study revealed sufficient genetic variability in the nightshade accessions, which can be exploited for crop improvement.

## 1. Introduction

Black nightshades (cultivated species from the genus Solanum section Solanum) are indigenous leafy vegetables that have the potential to improve food and nutritional security in Sub-Saharan Africa [1]. Indigenous leafy vegetables are a diverse group of unrelated species whose leaves are consumed mainly by rural communities [2]. These vegetables play a major role in household food security [2,3]. Human consumption of their leaves and fruits as food is widespread, especially in Africa and South-East Asia [4].

Genetic variations occur within varieties within species as well as between species, and it is these variations that cause genetic diversity. Some of these species may be phenotypically similar, posing a taxonomic challenge. Though they are morphologically similar, they differ genetically, which is why African nightshade has been reported to be genetically diverse [5]. Plant growth habits, stem color and ridging, leaf shape and pubescence and other phenotypic differences between species can be observed. Their flower and berry sizes significantly decrease during senescence, becoming fewer and smaller than usual. Broad leafed African nightshade cultivar (*Solanum scabrum*) is one of the most common and promising African nightshade species in Kenya, distinguished by its vigorously growing broad leaves and large purple berries. It varies in leaf size and plant height, but its leaf production remains higher than that of narrow-leafed species such as *Solanum villosum* and *Solanum eldoretianum*. One of the most widely distributed and consumed African nightshade species is *Solanum scabrum* [6]. Although nightshades have traditionally been regarded as inedible poisonous plants or troublesome agronomic weeds in Europe and the Americas [7,8,9], their status is completely different in western, eastern and southern Africa as well as India, Indonesia and China, where they have long been used as leafy herbs and vegetables, a source of fruits and dye, and for various medicinal uses as reported by [8,10]. 

The present study’s goal was to conduct a quantitative morphological characterization of available nightshade accessions with the specific goal of computing genetic variance components and using them to identify superior lines exhibiting desirable traits that can be used as breeding lines or recommended as cultivars. 

## 2. Results and Discussion 

### 2.1. Analysis of Variance

For all measured traits, there were highly significant (*p* < 0.001) differences between accessions (Table 1). The significant differences revealed a high degree of variability among the accessions that could be used to improve nightshade. 

### 2.2. Variation among Accessions

The quantitative morphological traits showed considerable mean variation, as presented in Table 2. There were higher variations among the accessions in plant height, the number of (leaves, fruits), fresh mass (fruit, leaf fresh and stem fresh mass) and biggest leaf area. Plant height varied from 43.50 to 66.83 cm with a mean of 55.54 cm. The tallest accessions were N5547, Timbali, N0096, ManTown and Nshad9. In contrast, accessions with the most numerous leaves were Timbali, Sharon, SNig2495, Nfarm, N5547, N0096, Ncampus and SABGA. The number of primary branches varied significantly (Table 2). Accessions with bigger stems were Nshad40, Scabrum, ManTown, SABGA, NigSN18 and SRetrflx, as stem diameter (mm) range between 8.03 mm (N0096) and 16.42 mm (Nshad40) with a mean of 10.82 mm. The number of fruits ranged from 82.67 (N5547) to 300.06 (Sharon) with a mean of 163.86; however, accessions with a high number of fruits were Sharon, Timbali, N0096, Nshad40, Ncampus, NigSN18, Nshad9 and SNig2495. 

Fruit fresh mass ranged from 18.74 g/plant (Nfarm) to 140.32 g/plant (SABGA) with a mean of 79.18 g/plant. The mean 54.72 g was observed in leaf fresh mass (g) as it varied from 37.85 to 74.87 g/plant, with Ncampus, Scabrum, Nshad40 and SABGA having high fresh mass. The mean stem fresh mass ranged from 52.20 g (NigSN18) to 122.97 g/plant (Scabrum) with a mean of 85.82 g/plant. The mean leaf area (cm^2^) of the smallest leaves ranged from 3.15 cm^2^ (Sharon) to 9.76 cm^2^ (Scabrum) with a mean of 5.80 cm^2^ and the mean leaf area (biggest leaf) ranged from 9.15 cm^2^ (Sharon) to 101.84 cm^2^ (Scabrum) with a mean of 31.43cm^2^. The number of days to flowering ranged between 26.33 (Nfarm and SNig2495) and 36.83 (Sharon), with a mean of 30.32. On average, the Nfarm and SNig2495 flowered earlier (approximately 26 days from transplanting) than Sharon (flowering late at approximately 37 days from transplanting). The mean number of days to fruit forming varied between 41.83 and 51.50 with a mean of 45.00 days from seedling transplanting. The number of days to fruit ripening ranged from 47.66 to 58.66, with a mean of approximately 52 days from transplanting. 

The analysis of variance also showed variation in fresh leaf yield per plant ranging from 301.60 g (Sharon) to 805.30 g (Scabrum), with a mean of 539.19 g/plant. The stem fresh yield ranged from 466.50 g (Sharon) to 1376.90 g (N5547) with a mean of 972.19 g/plant, while the fruit yield ranged from 173.40 g (Nfarm) to 1782.00 g (NigSN18), with a mean of 871.16 g/plant. The total biomass varied between 1044.00 g (Sharon) and 3347.20 g (ManTown), with a mean of 2437.37 g/plant. Finally, the harvest index in percentage varied between 19.60% (SRetrflx) and 38.87% (Nfarm), with a mean of 27.85% (Table 2). This study showed great variation among accessions in terms of the traits of importance and agronomic yield; however, a slightly significant variation was observed in leaf yield. Among the morphological characteristics that were measured for this present study, the biomass of leaf fresh mass (measured as an average leaf fresh mass g/plant), leaf area and the number of leaves and days to 50% flowering contribute to the amount of harvested biomass that can be used for consumption. 

In a study by [1], the traits of agronomic importance, such as leaf yield expressed as leaf fresh mass, leaf area, plant height, fruit mass and stem diameter, varied significantly between and within accessions used in their study. In contrast, in this present study, the findings are in agreement as it showed a significant variation in plant height, number of leaves, number of primary branches, number of fruits and fruit mass. In contrast, leaf fresh mass showed a slightly significant variation among accessions. The study revealed that accessions with the biggest leaf area (size) resulted in high leaf fresh mass, and these accessions are Scabrum, Nshad40 and ManTown. This agrees with the recommendations by [11] as well as [1], who stated that leaf yield in African nightshades should be considered in terms of leaf fresh mass as well as the number of leaves. The number of days to flowering influences the harvest in that of the onset of flowering, so late flowering allows for a prolonged period of harvesting. Flower removal as soon as they appear has been suggested to increase leaf yield and prolong harvesting periods as stated by [1,5,12]. From this present study, five of fifteen accessions (Nfarm, Ncampus, SNig2495, N0096 and Timbali) flowered early at the seedling stage (6 weeks from seed sowing in seedling trays); however, flowers were removed a day before seedling transplanting. 

### 2.3. Phenotypic Correlation for Quantitative Traits

There was a strong and positive correlation among some traits. The strength and nature of correlation among quantitative traits were analyzed. Correlation analysis varied significantly at a 0.05 probability level, with positive and negative correlations ranging from strong to moderate to weak. Positive and significant relationships were found between stem diameter, fruit fresh mass, leaf fresh mass, leaf dry mass, stem fresh mass, stem dry mass, small and largest leaf area and days to 50% flowering. Another strong positive correlation was observed between the number of leaves and the number of primary branches; leaf yield was also strongly correlated with stem yield per plant (r = 0.85). Negative and significant associations were observed for the number of leaves with stem diameter (r = −0.76), fruit fresh mass (r = −0.77), both small (r = −0.80) and biggest leaf area (r = −0.72), fruit fresh yield(r = −0.74) as well as total biomass (r = −0.82). 

Positive and moderate associations were found for the number of leaves and harvest index, the number of primary branches and the leaf chlorophyll content, the stem diameter and fruit yield, the fruit fresh mass and the largest leaf area ( r= 0.51), the stem dry mass and stem yield (r = 0.53). Negative moderate interactions were observed between the number of leaves and stem diameter (r = −0.50), the number of primary branches and leaf yield (r = −0.48) and the leaf chlorophyll content and days to 50% flowering (r = −0.52). Plant height, the number of primary branches, stem diameter, plant spread, dry leaf mass, stem fresh mass, smallest and largest leaf area and stem yield all had positive weak associations. Negative weak associations were observed for plant height, number of leaves, stem diameter, leaf chlorophyll content, plant spread, number of fruits per plant, leaf dry mass, days to 50% flowering, fruit forming and fruit ripening and stem yield per plant. 

When selecting desirable parental lines for yield improvement, correlation analysis is useful for determining traits that influence variation as well as interrelatedness [13]. The analysis findings revealed several correlations between the traits under consideration. Fruit yield and total biomass were positively and significantly correlated, whereas stem diameter, leaf fresh mass, stem fresh mass, leaf area and the number of leaves were positively and significantly correlated with stem fresh mass, biggest leaf area, leaf yield and total biomass. The primary branches, on the other hand, exhibited a negative and significant correlation with fruit fresh mass, small and biggest leaf area and total biomass per plant. These findings are consistent with those reported by [1,14]. Plant height was also found to have a weak positive relationship with stem fresh mass, stem dry mass, leaf yield and stem yield per plant. These positive correlations among plant height, number of leaves, plant spread, leaf fresh mass, fruit fresh mass, leaf area and number of days to 50% flowering imply that tall plants that flower later have larger stems, more time to accumulate photosynthesis and more time to spread and produce leaves with high fresh weight. A positive correlation between fresh mass and yield descriptors such as leaf area, plant height and stem indicates that such traits should be prioritized when selecting for high yield [1].

As reported in eggplant (*Solanum melongena*), positively correlated traits may have a common genetic and physiological basis [15]. The strong positive correlation suggests that indirect selection is possible; thus, selecting for one trait would result in the selection of another trait that is strongly correlated with the selected trait. As a result, a negative correlation between agronomic and yield-related traits indicates that traits with a high number of leaves may not be achieved simultaneously with stem diameter and leaf yield. This suggests that an increase in one would result in a decrease in the other [16]. When applying selection to a trait of interest, knowledge of correlation is essential to obtain the expected response of the other trait. Correlation estimates allow for the evaluation of one or more traits’ behavior using the performance of the other as a reference. There is a thorough understanding of indirect selection. This is especially true for traits that are difficult to quantify and are associated with low heritability [17]. Positively correlated traits imply gene linkage and pleiotropic effects [18,19,20]. Strong positive correlations among genotypes indicate that such traits are heritable and genetically controlled, implying that they can be transmitted to the desired genotypes [19].

### 2.4. Principal Component Analysis (PCA)

The first four principal components (PC1, PC2, PC3 and PC4) with eigenvalues greater than 1.00 explained 86.82 percent of the total variation for all traits. These were extracted and are shown in Table 3. The first principal component (PC1) accounted for 42.03 percent of total variation and had an eigenvalue of 9.25. Traits that contributed to this variation included both the largest and the smallest leaf area, stem diameter, stem yield and total biomass. The eigenvalue of the second PC was 4.41, and it accounted for 20.07 percent of the total variation. Days to 50% flowering, days fruit forming, leaf dry mass, harvest index, leaf fresh mass and plant spread were all components in this PC. The third PC accounted for 16.41% of total variation and had an eigenvalue of 3.61. The traits that contributed to this variation were leaf chlorophyll content, leaf yield, harvest index and leaf fresh mass. Finally, the fourth PC accounted for 8.31 percent of total variation and had an eigenvalue of 1.83, with plant height being the major contributor. 

Components with eigenvalues less than 1.00 were estimated in the principal component analysis, as explained by [20,21]. Component loadings of ± 0.03 are considered meaningful by [22], so they were selected. In the current study’s findings, the first four principal component axes explained 86.82 percent of the variation, indicating significant diversity among the traits, which agrees with [23,24] findings of similar variation. From the first component (9.25) to the fourth component (1.83), the eigenvalues decreased significantly. Though there are no clear guidelines for determining the significance of a character coefficient, one rule of thumb is to consider coefficients greater than 0.6 to have a large enough effect to be considered significant [25,26,27]. Small leaf area, largest leaf area, stem diameter, stem yield and total biomass were the traits that contributed the most to the first component, accounting for 42.03 percent of the total variation. This implies that selection in the first component should be centered on these characteristics. The first and second components showed the most variation among the accessions, with a cumulative variation of 62.10 percent, indicating a high degree of association among the traits [19].

### 2.5. Principal Component Biplot

**Figure 1:**
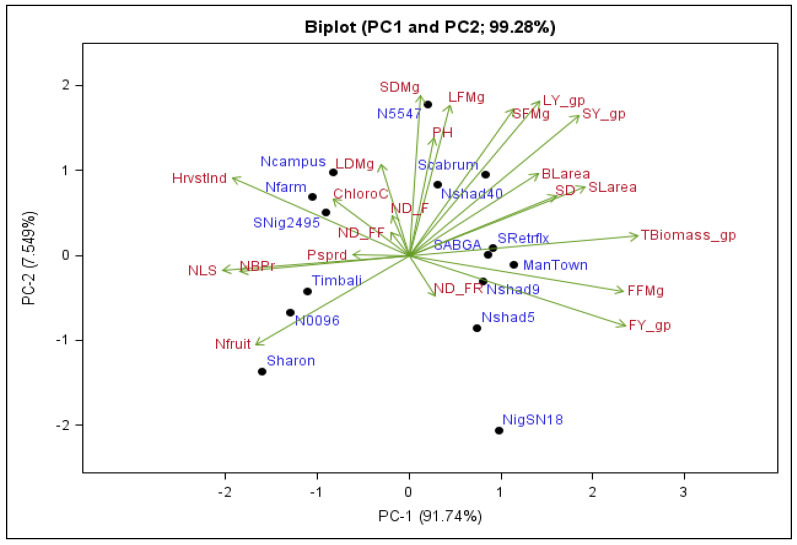
Principal component analysis biplot describing the overall variation among nightshade accessions estimated using 22 traits. PC—principal components 1 and 2. Black dots show the position of accessions in the quadrant and vector lines show how a trait influences the grouping of accessions.

A multidimensional biplot in Figure 1 illustrates the observed phenotypic diversity among the nightshade accessions under study. The biplot represents the relationship among phenotypic traits and accessions with the principal components. Traits with small angles between dimension vectors in the same direction have a high correlation in discriminating accessions [28]. Such relationships were found to be positively and significantly correlated in this study between leaf fresh mass and plant height, stem diameter and small leaf area. Accessions that excelled in a specific trait were plotted closer to the vector line and further in the direction of that vector. For example, stem diameter and small leaf area, leaf fresh mass and plant height were all positively and significantly correlated. This implies that selecting for small leaf area can be accomplished through selection for thicker and succulent stems, while selecting for leaf fresh mass can be accomplished through selection for tall plants. The biplot grouped the accessions based on their associations with phenotypic traits. Scabrum was associated with plant height and leaf fresh mass per plant, whereas Nshad40 was associated with stem fresh mass, stem yield and leaf yield per plant (Figure 1). 

### 2.6. Cluster Analysis

**Figure 2:**
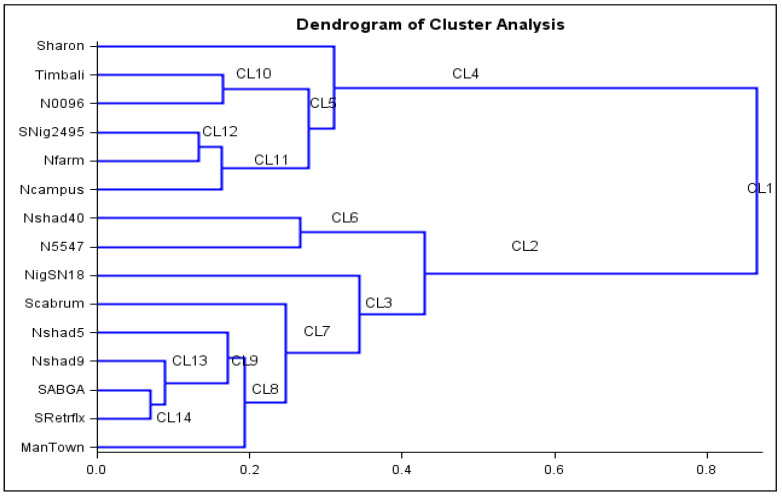
Cluster analysis showing phenotypic association among nightshade accessions. CL—cluster.

To evaluate the phenotypic relationships among accessions, the minimum distances between all pairs of individual accessions were constructed by plotting on a dendrogram (Figure 2). To gain a better understanding of the overall diversity of nightshade accessions, the data were analyzed using cluster analysis, which revealed the similarities between the genotypes. SRetrflx, SABGA and Nshad9 had the lowest dissimilarity index (0.00) (Table 5). Cluster 1 accessions had the highest dissimilarity index of 0.8652 and the mean distance between observations was 1245.994. Cluster analysis was used in this study and grouped the accessions into 14 clusters. Cluster 1 was a component of all accessions attributed to the following clusters: 4, 5, 10, 11 and 12. These clusters contained accessions that are not high yielding and are early flowering. Cluster 2 was a component of clusters 3, 6, 7, 8, 9, 13 and 14, and all accessions in these clusters performed well in terms of agronomic and yield-related traits, with three accessions (N5547, Nshad40 and Scabrum) showing late flowering. Cluster 6 included two of the five late-flowering accessions (Nshad40 and N5547).

### 2.7. Genetic Parameters

For all traits, the results revealed significant genotypic and phenotypic variances among the accessions (Table 4). The genetic variance ranged from 7.20 for the number of primary branches to 21,556, 8.63 for the total biomass per plant, while the phenotypic variance ranged from 14.72 for the number of primary branches to 16,322, 09.40 for the total biomass per plant. The estimates for the phenotypic coefficient of variation (PCV) were higher than those for the genetic coefficient of variation for all traits, indicating that environment influences trait expression. GCV values ranged from 5.70 percent for leaf yield per plant to 172.92 percent for the largest leaf area, while PCV values ranged from the biggest leaf area to fruit fresh mass. The results of broad-sense heritability revealed that the traits had high significant estimates. The estimates for leaf yield per plant ranged from 2.28 percent to 70.59 percent for the smallest leaf area. The expected genetic advance as a percentage of the mean (GAM) ranged from 1.77 percent for leaf yield to 254.25 percent for the largest leaf area, followed by 142.40 percent for the smallest leaf area. 

The analysis of trait variability and association of a trait with other traits contributing to crop yield would be important in planning a successful breeding program [29]. The observed variability is a composite of genetic and environmental effects, of which only the former is inherited. Estimates of heritability, on the other hand, do not provide an idea of the expected gain in the next generation and must be considered in conjunction with estimates of genetic advance, the change in mean value among successive generations [14,30,31,32].

The magnitude of GCV and PCV values indicate the degree of variability shown by different parameters. GCV revealed that the extent of genetic variability in the characters ranged from moderate to high. This suggests that there was a greater environmental influence on the expression of these traits, and selection may be effective in improving nightshade. Furthermore, for all traits, GCV values were low in magnitude when compared to PCV. This suggests that direct selection would be ineffective in these traits. These findings are consistent with [33] on poppy and [34] on lemongrass. Although the genotypic coefficient of variation revealed the extent of genetic variability present in accessions for various traits, it does not provide a complete view of heritable variation. Heritable variation is useful for permanent genetic improvement [30,31,35]; however, the extent of heritable variation cannot be ascertained using GCV alone. Thus, heritability estimates indicate the efficacy with which selection could be expected to exploit existing genetic variability, as stated by [36,37]. 

Phenotypic and genotypic variances in a crop population are important for successful plant breeding. Broad-sense heritability (H^2^) is expressed as the percentage of the ratio between the genotypic variance and phenotypic variance. Heritability is classified as low (below 30%), medium (30–60%) and high (above 60%); genetic advance (as a percentage of mean) is classified as low (<10%), moderate (10–20%) and high (>20%) [38]. In the current study, the smallest leaf area had a high heritability in the broadest sense. The highest heritability estimates were for the smallest leaf area (70.59%), followed by a relatively high moderate heritability for the biggest leaf area (50.94%) (Table 4). The heritability estimates for the number of leaves, number of primary branches, leaf dry mass, biggest leaf area, days to 50% flowering, days to fruit forming and days to fruit ripening were 49.79, 48.88, 35.26, 50.94, 42.18, 48.18 and 36.84%, respectively; all other traits had low estimates. 

Though heritability estimates may not provide clear predictability of breeding values, they are usually more useful than simple heritability values in predicting the resultant effect of selecting the best individual. Heritability coupled with genetic advance is a significant factor in predicting the resultant effect for selecting the best individuals [38]. Harvest index was low in GA with moderate GAM, revealing that the trait was governed by additive gene action and low heritability, revealing that this trait was influenced by environmental influence. For the number of leaves and largest leaf area, there was moderate broad sense heritability combined with high genetic advance, indicating that the traits are primarily controlled by the additive type of genes; however, for primary branches, leaf dry mass, days to 50% flowering, days to fruit forming and days to fruit ripening, moderate heritability was observed, along with low GA and high GAM (Table 4). As a result of these findings, it was determined that the action of additive genes involved in the expression of these traits was important. Low heritability combined with low genetic advance indicates non-additive gene effects, and thus a low genetic gain from selection is expected [39,40]. Plant height, stem diameter, leaf chlorophyll content, leaf fresh mass per plant and harvest index all had low heritability and low genetic advance, indicating the involvement of non-additive genes in the expression of these traits. The current study’s findings are consistent with previous reports on amaranths by [41,42,43], who discovered a low heritability (16.09%) with a low genetic advance (0.16) for seed weight in Mustard. In contrast, Saifullah [44] discovered a high heritability (65.03%) with a low genetic advance (0.31%). 

The heritability for the number of fruits, fruit fresh mass, stem yield, fruit yield and total biomass was low, despite high genetic advance (Table 4). Such traits are more influenced by the environment, making selection ineffective for them as indicated by [45,46]. Furthermore, traits that exhibit high heritability with moderate or low genetic advance can be improved by inter-mating the superior accessions of the segregating population developed from multiple crosses and the desirable genes can be accumulated in the lines as stated by [46,47]. On the other hand, an association of high heritability with high genetic advance is an indication of additive gene effects and consequently, a high genetic gain from selection would be anticipated [14,48]. 

Results of this study suggested that if the top performing 5% were selected as parents, the mean biggest leaf area, smallest leaf area, fruit fresh mass, number of leaves, number of primary branches and days to 50% flowering for progenies would improve by 79.91 cm^2^, 8.26 cm^2^, 93.61 g/plant, 88.06, 16.88 g/plant and 9.97 days, respectively. When combined with heritability estimates, the estimate of genetic advance is more useful as a selection tool [30,31,38]. Estimating genetic progress aids in understanding the type of gene action involved in the expression of various polygenic traits [20,30,31]. 

High GA values indicate additive gene action, whilst low values indicate non-additive gene action [20,30,31,49]. As a result, the heritability estimates would be useful if they are accompanied by a high genetic advance. In the present study, the highest expected GAM was observed for the largest leaf area, smallest leaf area, fruit fresh mass, number of leaves, number of fruits per plant, leaf dry mass, number of primary branches, fruit yield, leaf chlorophyll content, days to 50% flowering and leaf fresh mass (Table 4), respectively. The authors of [14,46,50] reported similar findings of high genetic advance over a mean number of leaves and other related traits. High GAM values indicate that these traits are simply inherited; the heritability is most likely due to additive gene effects and selection may be effective in early generations for these traits [30,31,32]. As a result, selecting for traits with high GAM will improve the performance of nightshade accessions for these traits.

## 3. Materials and Methods

### 3.1. Plant Materials and Experimental Design and Treatments

Fifteen Nightshade accessions were evaluated in the study. The accessions came from a variety of sources within South Africa. Table 5 below provides detailed information on each accession. The study was set up in a randomized complete block design (RCBD) replicated three times. Each accession was planted in a 2.5-m long 5-row plot with inter-row and intra-row spacing of 50 cm. The experiment was conducted over two summer growing seasons (January–April 2020 and December 2020–February 2021).

### 3.2. Description of the Research Site, Trial Establishment and Maintenance 

Accessions were planted in seedling trays in a 40% black shade-net house (i.e., the nethouse allowed 60% of solar radiation to pass through to the plants) at the North-West University (NWU) Mafikeng, South Africa. Plants were transplanted directly onto the field of the NWU research garden five weeks after planting. The experimental site is located at 25. 810 S and 25. 630 E, with an altitude of 1276 m. The experiment was carried out during the summers of 2020/21 (January to April 2020 and December to February 2020–2021). The precipitation and average temperature for both cropping seasons in the NWU Mafikeng campus are presented in Table 6. The total supplementary irrigation in the first season was 720 mm, while it was 660 mm in the second season. During the study period, no fertilizer was used. Malasol^®^ 2.5 E.C (active ingredient: Mercaptothion (Organophosphate) 500 g/L) (Benmore, South Africa) was sprayed regularly to control black aphids, and Efekto Redspidercide (active ingredient: Tetradifon 81.0 g/L) was sprayed regularly to control red spider mites, using a knapsack sprayer based on the manufacturer’s recommendation for the closely related Solanaceous crop tomato, as there are no recommendations for nightshade yet. Hand-hoeing was used for manual weeding twice per growing season. Soil samples were collected from the study site and sent to CEDARA, which is part of the KZNDARD and has soil analytical facilities, for soil analytical service and analyses. Table 7 presents the results of the soil physical and chemical characteristics within the top 30 cm. Based on the South African soil taxonomic system, the soil is classified as Coega form. 

### 3.3. Data Collection

In each plot, data for morphological traits were collected from six randomly selected plants per accession. For this study, the following quantitative traits were assessed: plant height was measured in cm using a meter from the base of the tip of the main stem, the number of leaves was recorded by physically counting mature leaves per plant, the number of primary branches (branches attached to the main stem of the plant) was counted using a digital veneer caliper, the stem diameter in millimeters was measured at the base of the first internode from all data plants and the leaf chlorophyll content index nm was measured using the CCM-200 PLUS GPS CHLOROPHYLL CONTENT (Winn Avenue, Hudson, United State of America). The plant spread in centimeters was measured using a tape measure graduated in millimeters, centimeters and meters, leaf fresh and dry mass in grams, stem fresh and dry mass in grams was also measured, leaf length and leaf width were taken from a total of 12 leaves per net plot for both small (the 3rd leaf from the top of the main stem) and the biggest leaf was recorded in centimeters using a meter ruler. The leaf area (cm^2^) was calculated using the recorded leaf length and width as follows: Leaf area (cm^2^) = LL ∗ LW ∗ 0.75, where 0.75 is a constant, as determined by the non-destructive length * width method described by [51,52]. The number of days from transplanting to when approximately half of the plants in each plot flowered, the number of days from transplanting to fruit-forming and the number of days from transplanting to fruit ripeness were all recorded. After seedling transplantation, the field was monitored daily to determine phenological attributes such as the number of days to 50% flowering, days to fruit forming and matured fruit ripeness.

The yield was determined as fresh yield (total biomass) in grams per plant and also per plot. It was portioned into leaf, stem and fruit yield. Harvesting of leaves and young shoots was performed once at trial termination. Harvesting was performed by pinching off tender and new shoot leaves early in the morning to obtain fresh weight and avoid weight loss during the day. All plants in the net plots were harvested; however, the trial was harvested only once for research purposes. At the harvesting stage, the fresh weight, which included leaves and tender new shoots, as well as the number of fruit and fruit weight, were recorded. Nightshade fruits were counted using two techniques: manually and/or with a seed counting machine. Small to medium fruits were counted using a Numigral CHOPIN machine (the Numigral Analyzer accurately counts 1000 grains using optical sorting) (Marcellin Berthelot, Villeneuve-la-Garenne Cedex, France); large fruits were manually counted. Total biomass was computed as the sum of total above-ground mass in grams per plant, and harvest index was calculated as the ratio of leaf fresh mass to total above-ground mass in percentage. 

### 3.4. Data Analyses

All statistical analysis was performed using SAS Version 9.4 [53]. Analysis of variance (ANOVA) was performed and Tukey’s honest significant difference (HSD) test [54] was used to separate means at *p* < 0.05. The similarities and dissimilarities were determined using hierarchical clustering analysis of the unweighted pair group method of arithmetic (UPGMA) mean. A principal component analysis biplot was used to demonstrate the associations between accessions based on observed traits. To describe the relationship between the morphological traits, Pearson’s correlation analysis was used. 

Heritability in the broad sense was calculated using a formula by [30,31,55] as follows;

(1) $$H^{2}=\frac{\sigma^{2}g}{\sigma^{2}p}$$

where $H^{2}\;$= broad-sense heritability; $\sigma^{2}g$ is the genotypic variance; $\sigma^{2}p$ is the phenotypic variance were obtained from the analysis of variance table. The (phenotype) is the mean of genotype across *m* trials (environments) and *r* replicates per trial. This has variance

(2) $$\sigma^{2}p=\sigma^{2}g+\frac{\sigma^{2}ge}{m}+\frac{\sigma^{2}}{rm}$$

where $\sigma^{2}ge$ is the genotype–environment interaction variance and $\sigma^{2}$ is the residual error variance [56,57].

Heritability was categorized as low, moderate and high following [58,59] as follows; as low (<10%), moderate (10–20%) and high (>20%). Estimation of variance components; the genotypic and phenotypic variance was calculated based on equations by [30,31,60] using;

(3) $$\mathrm{Genotypic}\;\mathrm{variance}\;\sigma^{2}g=\frac{MS1-MS2}{rs}\;\mathrm{and}\;\mathrm{phenotypic}\;\mathrm{variance}:\;\sigma^{2}p=\frac{MS1}{rs}$$

where *MS*1 is the mean square due to genotype, *MS*2 is the mean square for species by season interaction, *S* = season and *r* is the number of replications. The mean values were used for genetic analyses to determine the genetic coefficient of variation (GCV); phenotypic coefficient of variation (PCV) was computed as described by [30,31,61] and expressed as a percentage as follows:

(4) $$\mathrm{Genotypic}\;\mathrm{covariance}\;\left(\mathrm{GCV}\;\%\right)=\frac{\sqrt{\sigma^{2}g}}{X}\times 100\;\mathrm{and}\;\mathrm{phenotypic}\;\mathrm{covariance}\;\left(\mathrm{PCV}\;\%\right)=\frac{\sqrt{\sigma^{2}p}}{X}\times 100;$$

where $\sigma^{2}p$ = phenotypic variation and $\sigma^{2}g$ = genotypic variation; *X* = Grand mean of the characters measured. Genetic advance (GA) was calculated by selecting 5% of the superior accessions and calculated using the following formula from [30,31,62] as

(5) $$\mathrm{GA}=k\cdot \sigma^{2}p\cdot h^{2}$$

where GA= expressed genetic advance, $\sigma^{2}p$ = square root of phenotypic variance and *k*: constant = selection differential (2.06) at 5% selection intensity. GA as % of mean (GAM) = $\frac{GA}{X}\times 100$.

## 4. Conclusions

In the current analyses of phenotypic diversity of nightshade accessions using both agronomic and quantitative morphological traits, a variety of observations were made. For all traits, all 15 accessions varied significantly from others, indicating the presence of significant variation among them. Correlation studies are important in breeding programs since they help breeders understand the interrelatedness among various agronomic and morphological traits and use the results for selection during breeding work. This study also revealed that some morphological traits distinguished accessions more effectively than others and that quantitative traits are important in assessing genetic diversity within nightshade accessions and could be used to select relevant accessions to be incorporated into breeding programs for crop improvement; however, for all of the traits assessed, the phenotypic variance and phenotypic coefficient of variation were greater than the corresponding genotypic variance and genotypic coefficient of variation. Considering all of the traits associated with high vegetable yield and late maturity, the accessions Scabrum, Nshad40 and NigSN18 have the potential to contribute to the development of improved late-flowering varieties that remain in the vegetative phase for longer in South African nightshade breeding. 

## Figures and Tables

**Table 1:** Analysis of variance and significance tests for nightshade accessions for combined data of two seasons.

	Mean Squares						
Source of Variance	SEASON	Rep	Access	S*Access	Residual Error	CV%	GM
DF	1	2	14	14	508		
PH cm	37,500.00	2272.20	1541.61	972.97	170.12	23.48	55.54
NLS	386,082.81	126,599.01	28,148.89	6127.77	3822.37	50.35	122.77
NBPr	18.51	31.57	56.33	13.14	5.70	30.93	7.72
SD mm	941.84	13.59	152.50	105.78	57.79	70.19	10.83
ChloroC nm	171.92	682.19	857.30	375.56	120.26	39.69	27.63
Psprd mm	180,950.41	332,508.37	52,391.55	38,209.14	8513.04	21.15	436.26
Nfruit	1,803,360.06	16,647.53	145,177.30	75,054.82	17,846.65	81.52	163.87
FFM g	471,865.09	24,732.61	73,300.68	31,490.02	4823.89	87.72	79.18
LFM g	41,018.36	3167.15	4806.58	2650.23	1434.49	69.21	54.73
LDM g	4411.95	269.51	337.36	109.12	91.66	71.36	13.42
SFM g	209,762.49	54,980.50	15,658.43	16,752.00	3511.77	69.05	85.82
SDM g	16,668.55	2718.57	456.95	617.37	179.64	69.30	19.34
SLarea cm^2^	22.97	38.43	154.27	17.61	57.79	35.14	5.80
BLarea cm^2^	998.11	2009.37	23,268.50	5545.11	433.21	66.22	31.43
ND_F	4611.26	270.06	481.20	148.12	12.29	11.56	30.32
ND_FF	10,454.40	131.40	357.28	91.40	11.75	7.62	45.00
ND_FR	1092.26	168.80	314.17	112.83	6.63	4.97	51.80
**Source of Variance**	**SEASON**	**Rep**	**Access**	S*Access	**Residual Error**	**CV%**	**GM**
**DF**	**1**	**2**	**14**	**14**	**58**		
LY_gp	1,224,365.41	64,213.01	90,383.08	83,521.08	43,006.78	34.95	593.19
SY_gp	5,775,868.26	1,220,950.45	419,336.89	338,286.70	136,080.38	37.91	972.99
FY_gp	14,048,539.10	545,708.29	1,944,116.32	1,003,041.28	147,601.51	44.10	871.17
TBiomass_gp	52,677,869.68	4,210,043.66	3,895,404.18	2,601,992.42	693,867.4	34.17	2437.36
HrvstInd%	785.40	289.58	224.65	101.48	31.46	20.13	27.85

DF—degree of freedom, Rep—replicate, Access—accession, S*Access—season by accession interaction, CV%—coefficient variation percentage, GM—grand mean. PH—plant height, NLS—number of leaves, NPBr—number of primary branches, SD—stem diameter, ChloroC—leaf chlorophyll content, Psprd—plant spread, Nfruit—number of fruits, FFM—fruit fresh mass, LFM—leaf fresh mass, LDM—lead dry mass, SFM-stem fresh mass, SDM—stem dry mass, SLarea—smallest leaf area, BLarea—biggest leaf area, ND_F—number of days to 50% flowering, ND_FF—days to fruit forming, ND_FR—days to fruit repines. LY_gp—leaf yield in gram per plant, SY_gp—stem yield in gram per plant, FY_gp—fruit yield in gram per plant, TBiomass_gp—total biomass in grams per plant, HrvstInd—harvest Index per-centage.

**Table 2:** Variation for 22 traits in 15 nightshade accessions and means under combined analysis.

Accession	PH (cm)	NLS	NBPr	SD (mm)	ChloroC nm	Psprd mm	Nfruit	FFM (g)	LFM (g)	LDM (g)	SFM (g)	SDM (g)
**ManTown**	61.33 ab	94.67 df	6.41 de	12.16 ab	23.59 c	418.89 bd	136.47 c e	123.41 a	48.82 ac	10.38 bc	83.70 ad	16.67 bc
**NigSN18**	43.50 d	104.14 de	7.78 ad	11.58 ab	24.95 bc	443.61 ac	161.42 be	111.17 ab	43.01 bc	10.68 bc	52.20 d	11.34 c
**SRetrflx**	56.11 bc	101.28 de	6.97 be	11.57 ab	26.50 bc	453.33 ac	116.42 de	120.97 ab	49.21 ac	10.89 bc	91.15 ad	19.09 ac
**Nshad9**	59.83 ac	101.19 de	8.31 ac	10.82 ab	25.71 bc	457.22 ac	155.39 be	130.68 a	52.85 ac	11.12 bc	94.61 ad	19.79 ac
**Nshad5**	49.61 cd	113.31 be	5.97 de	10.18 b	28.25 bc	359.17 d	98.39 e	108.15 ab	50.55 ac	12.01 bc	81.72 ad	17.53 ac
**N5547**	66.83 a	138.22 ae	7.61 ad	10.94 ab	25.29 bc	405.28 bd	82.67 e	67.20 ac	59.83 ac	13.34 ac	109.87 ab	22.72 ab
**Nshad40**	57.89 ac	90.39 ef	7.08 cd	16.42 a	22.20 c	460.00 ac	210.67 ad	100.65 ac	71.33 ab	17.82 ab	114.10 ab	21.22 ac
**Ncampus**	51.83 bd	127.08 be	8.86 ab	9.83 b	39.34 a	469.72 ab	188.47 be	24.03 d	74.88 a	15.97 ac	82.07 ad	21.32 ac
**Nfarm**	53.67 bd	150.08 ac	9.31 a	9.84 b	33.69 ab	459.44 ac	119.36 de	18.74 d	54.67 ac	14.20 ac	81.70 ad	19.16 ac
**N0096**	61.63 ab	135.06 be	8.33 ac	8.03 b	25.62 bc	390.56 cd	241.94 ac	30.88 d	39.24 c	9.62 c	57.77 cd	16.66 bc
**Scabrum**	56.81 ac	85.47f	5.06 e	12.54 ab	22.92 c	433.06 bd	122.03 de	107.43 ab	73.49 a	19.91 a	122.97 a	27.75 a
**SNig2495**	50.39 cd	140.33 ad	8.81 ab	9.39 b	32.68 ab	446.25 ac	150.97 be	26.36 d	55.55 ac	12.59 ac	73.32 bd	18.16 ac
**Sharon**	45.28 d	153.92 ab	8.39 ab	9.33 b	23.25 c	516.94 a	300.06 a	45.14 cd	49.31 ac	17.11 ac	76.85 ad	19.09 ac
**SABGA**	56.14 bc	121.14 be	7.53 ad	11.63 ab	32.60 ab	432.22 bd	115.81 de	140.32 a	60.31 ac	13.98 ac	105.00 ad	21.28 ac
**Timbali**	62.31 ab	185.36 a	9.42 a	8.18 b	27.86 bc	398.33 bd	257.94 ab	32.58 d	37.85 c	11.65 bc	60.31 cd	18.33 ac
**HSD**	**	***	***	**	**	**	***	***	**	*	**	**
**Fpr**	<0.0001	<0.0001	<0.0001	00.0010	<0.0001	<0.0001	<0.0001	<0.0001	<0.0001	<0.0001	<0.0001	0.0016
**GM**	55.54	122.77	7.72	10.83	27.63	436.27	163.87	79.18	54.73	13.42	85.82	19.34
**Accession**		**SLarea** **(cm^2^)**	**BLarea** **(cm^2^)**	**ND_F**	**ND_FF**	**ND_FR**	**LY_gp**	**SY_gp**	**FY_gp**	**TBiomass_gp**	**HrvstInd** **(%)**	
**ManTown**		6.10 b	51.61 c	28.33 bc	43.83 cf	52.17 cf	651.90 ab	1187.30 ac	1507.90 ab	3347.20 a	20.77 ed	
**NigSN18**		6.50 b	22.36 de	28.50 bc	44.67 be	53.33 be	532.60 ab	765.10 ac	1782.00 a	3079.70 ab	21.86 ce	
**SRetrflx**		6.68 b	33.26 d	30.16 b	45.83 bd	52.33 cf	611.70 ab	1206.60 ac	1354.20 ac	3172.60 ab	19.60 e	
**Nshad9**		6.24 b	24.66 de	27.83 bc	43.83 cf	51.67 df	612.50 ab	1086.90 ac	1363.70 ac	3063.10 ab	21.29 ed	
**Nshad5**		6.14 b	23.19 de	27.83 bc	42.83 ef	49.17gh	580.20 ab	956.10 ac	1433.70 ac	2970.00 ac	26.30 be	
**N5547**		6.64 b	23.40 de	35.33 a	51.50 a	55.33 b	685.50 ab	1376.90 a	657.10 ce	2719.50 ad	26.47 be	
**Nshad40**		9.72 a	70.31 b	36.50 a	47.00 b	53.50 bd	679.00 ab	1192.80 ac	879.80 be	2751.60 ac	29.42 ae	
**Ncampus**		4.14 c	20.49 de	28.17 bc	43.67 df	47.67h	718.60 ab	911.60 ac	228.20 e	1858.40 ad	37.68 ab	
**Nfarm**		4.00 c	14.30 e	26.33 c	41.83f	48.17h	612.00 ab	856.80 ac	173.40 e	1642.20 bd	38.87 a	
**N0096**		3.38 c	10.17 e	29.83 b	41.83f	51.17fg	425.80 ab	615.30 bc	304.80 e	1346.00 cd	30.41 ae	
**Scabrum**		9.77 a	101.84 a	34.67 a	46.00 bd	53.83 bc	805.30 a	1252.00 ab	1132.70 ad	3190.10 ab	30.19 ae	
**SNig2495**		4.41 c	20.63 de	26.33 c	41.83f	48.17h	573.40 ab	880.30 ac	289.80 e	1743.50 ad	33.22 ac	
**Sharon**		3.16 c	9.16 e	36.83 a	51.50 a	58.67 a	301.60 b	466.50 c	275.90 e	1044.00 d	29.39 ae	
**SABGA**		6.53 b	35.13 cd	27.83 bc	42.33 ef	51.33 ef	622.30 ab	1161.50 ac	1331.70 ac	3115.50 ab	21.00 ed	
**Timbali**		3.52 c	10.99 e	30.33 b	46.50 bc	50.50fg	485.50 ab	679.10 ac	352.50 de	1517.20 bd	31.33 ad	
**HSD**		**	***	*	***	***	*	**	***	***	***	
**Fpr**		<0.0001	<0.0001	<0.0001	<0.0001	<0.0001	0.0250	0.0013	<0.0001	<0.0001	<0.0001	
**GM**		5.80	31.43	30.32	45.00	51.80	593.19	972.99	871.16	2437.37	27.85	

PH—plant height, NLS—number of leaves, NPBr—number of primary branches, SD—stem diameter, ChloroC—leaf chlorophyll content, Psprd—plant spread, Nfruit—number of fruits, FFM—fruit fresh mass, LFM—leaf fresh mass, LDM—lead dry mass, SFM-stem fresh mass, SDM—stem dry mass, SLarea—smallest leaf area, BLarea—biggest leaf area, ND_F—number of days to 50% flowering, ND_FF—days to fruit forming, ND_FR—days to fruit repines. LY_gp—leaf yield in gram per plant, SY_gp—stem yield in gram per plant, FY_gp—fruit yield in gram per plant, TBiomass_gp—total biomass in grams per plant, HrvstInd—harvest Index percentage, and Fpr—F probability (*p*-value), HSD—Honestly Significant difference at 5% probability level, GM—grand mean. * *p* < 0.05, ** *p* < 0.01, *** *p* < 0.001. Tukey pairwise comparisons analyzed at *p* < 0.05 significant level. Means within columns that do not share a letter are significantly different.

**Table 3:** Principal component analysis for 22 quantitative traits indicating eigenvectors, eigenvalues and proportion of variation explained with the four PCs axes.

	Eigenvectors			
Characters	PC1	PC2	PC3	PC4
PH (cm)	0.08	0.01	0.05	**0.65**
NLS	−0.27	0.12	0.01	**0.24**
NBPr	−0.27	0.06	0.08	0.00
SD (mm)	**0.28**	0.06	−0.04	−0.18
ChloroC	−0.14	−0.02	**0.42**	−0.14
Psprd (mm)	−0.02	0.25	−0.07	−0.46
Nfruit	−0.19	0.20	−0.24	−0.10
FFM g/plant	**0.26**	−0.22	−0.14	−0.09
LFM g/plant	0.19	0.26	**0.27**	−0.16
LDM g/plant	0.12	**0.40**	0.07	−0.20
SFM g/plant	0.27	0.19	0.11	0.11
SDM g/plant	0.17	**0.31**	0.18	**0.20**
SLarea cm^2^	**0.31**	0.02	−0.01	−0.05
BLarea cm^2^	0.28	0.11	0.04	−0.06
ND_F	0.11	0.34	−0.29	0.13
ND_FF	0.05	0.28	−0.32	0.17
ND_FR	0.10	0.18	**−0.44**	0.03
LY_gp	0.24	0.02	**0.32**	0.06
SY_gp	**0.28**	−0.04	0.15	**0.21**
FY_gp	0.22	−0.31	−0.14	−0.15
TBiomass_gp	0.28	−0.22	−0.00	−0.03
HrvstInd (%)	−0.16	**0.28**	**0.28**	−0.03
Eigenvalue (explained variance)	9.25	4.41	3.61	1.83
Proportion of total variance (%)	42.03	20.07	16.41	8.31
Cumulative variance (%)	42.03	62.10	78.51	86.82

PH—plant height, NLS—number of leaves, NPBr—number of primary branches, SD—stem diameter, ChloroC—leaf chlorophyll content, Psprd—plant spread, Nfruit—number of fruits, FFM—fruit fresh mass, LFM—leaf fresh mass, LDM—lead dry mass, SFM-stem fresh mass, SDM—stem dry mass, SLarea—smallest leaf area, BLarea—biggest leaf area, ND_F—number of days to 50% flowering, ND_FF—days to fruit forming, ND_FR—days to fruit repines. LY_gp—leaf yield in gram per plant, SY_gp—stem yield in gram per plant, FY_gp—fruit yield in gram per plant, TBiomass_gp—total biomass in grams per plant, HrvstInd—harvest Index percentage, and PC—principal components 1, 2, 3 and 4, respectively.

**Table 4:** Estimation of genetic parameters for agronomic and yield contributing traits of 15 nightshade accessions selected at 5% selection intensity.

Variables	Mean Range	X	*σ* ^2^ *g*	*σ* ^2^ *p*	H^2^ (%)	GCV (%)	PCV (%)	GA at 5%	GAM (%)
PH cm	43.50–66.83	55.54	94.77	609.62	15.55	17.53	44.45	7.91	14.24
NLS	85.47–185.36	122.77	3670.19	7371.14	49.79	49.35	69.93	88.06	71.73
NBPr	5.06–9.41	7.72	7.20	14.72	48.88	34.75	49.70	3.86	50.05
SD mm	8.03–16.42	10.83	7.79	70.31	11.08	25.79	77.50	1.91	17.68
ChloroC	22.20–39.34	27.63	80.29	288.12	27.87	32.43	61.43	9.74	35.27
Psprd mm	359.17–515.94	436.27	2363.73	22,888.89	10.33	11.14	34.68	32.19	7.38
Nfruit	82.67–300.06	163.87	11,687.08	52,188.94	22.39	65.97	139.41	105.39	64.31
FFM g	18.74–140.32	79.18	6968.44	23,517.40	29.63	105.43	193.68	93.61	118.22
LFM g	37.85–74.88	54.73	359.39	1923.59	18.68	34.64	80.14	16.88	30.84
LDM g	9.62–19.91	13.42	38.04	107.88	35.26	45.96	77.39	7.54	56.22
SLarea cm^2^	3.16–9.77	5.80	22.78	32.27	70.59	82.28	97.95	8.26	142.40
BLarea cm^2^	9.16–101.84	31.43	2953.90	5798.66	50.94	172.92	242.28	79.91	254.25
ND_F	26.33–36.83	30.32	55.52	131.62	42.18	24.57	37.84	9.97	32.88
ND_FF	41.43–51.50	45.00	44.32	91.97	48.18	14.79	21.31	9.52	21.15
ND_FR	47. 67–58.67	51.80	33.56	91.08	36.84	11.18	18.42	7.25	13.98
LY_gp	301.60–805.30	593.19	1143.67	50,072.00	2.28	5.70	37.72	10.53	1.77
SY_gp	466.50–1376.90	972.99	13,508.37	205,331.78	6.58	11.95	46.57	61.41	6.31
FY_gp	173.40–1782.00	871.16	156,845.84	682,966.73	22.97	45.46	94.86	390.97	44.88
TBiomass_gp	1044.00–3347.60	2437.37	215,568.63	1,632,209.40	13.07	19.05	52.42	347.59	14.26
HrvstInd (%)	19.60–38.88	27.85	20.53	76.51	26.83	16.27	31.41	4.83	17.36

PH—plant height, NLS—number of leaves, NPBr—number of primary branches, SD—stem di-ameter, ChloroC—leaf chlorophyll content, Psprd—plant spread, Nfruit—number of fruits, FFM—fruit fresh mass, LFM—leaf fresh mass, LDM—lead dry mass, SFM-stem fresh mass, SDM—stem dry mass, SLarea—smallest leaf area, BLarea—biggest leaf area, ND_F—number of days to 50% flowering, ND_FF—days to fruit forming, ND_FR—days to fruit repines. LY_gp—leaf yield in gram per plant, SY_gp—stem yield in gram per plant, FY_gp—fruit yield in gram per plant, TBiomass_gp—total biomass in grams per plant, HrvstInd—harvest Index percentage, and X—grand mean, *σ*^2^*g*—genotypic variance, *σ*^2^*p*—phenotypic variance, H^2^, PCV%—phenotypic coefficient of variation, GCV%—genotypic of variation, GA—genetic advance, GAM%—genetic advance as mean percentage

**Table 5:** Fifteen nightshade accessions evaluated during the study.

Entry No.	Given Codes	Accessions	Source of Origin in South Africa
1	ManTown	ManTown	ARC
2	NigSN18	NigSN18	ARC
3	*SRetrflx*	*Solanum retroflexum*	ARC
4	Nshad9	Nshad9	ARC
5	Nshad5	Nshad5	ARC
6	Nshad40	Nshad40	ARC
7	Scabrum	*Solanum scabrium* (Unizulu)	University of Zululand
8	SNig2495	*S. nigrum* 2495	DAFF
9	Ncapmus	Nshad NWU	NWU Mafikeng-campus
10	Nfarm	Nshad NWU	NWU Farm
11	Timbali	NshadTimbali	Komatiport- Mpumalanga
12	N0096	Nshad 0096	Naas- Mpumalanga
13	N5547	N5547	ARC
14	SABGA	SABGA	ARC
15	Sharon	Sharon	ARC

NWU—North-West University, ARC—Agricultural Research Council, DAFF—Department of Agriculture, Forestry and Fisheries.

**Table 6:** Weather conditions at NWU Mafikeng garden.

Month	Year	Minimum	Maximum	Rainfall (mm)	Relative Humidity (%) Average
		Temperature (°C)			
Season 1					
January	2020	20	30	179.3	51
February	2020	20	30	95.7	49
March	2020	18	29	97.2	45
April	2020	15	26	59.2	45
November	2020	20	30	229.5	46
Season 2					
December	2020	20	29	168.2	52
January	2021	17.4	30.8	335.8	58
February	2021	16.8	29.5	259.3	63

Source: South African Weather Service (SAWS) records.

**Table 7:** Physical and chemical characteristics of the field trial soil.

Soil Properties	2019/2020	2020/2021
Sample density g/mL	1.14	1.17
P mg/L	1	1
K mg/L	244	279
Ca mg/L	2961	1857
Mg mg/L	680	478
Zn mg/L	0.1	0.5
Mn mg/L	14	16
Cu mg/L	2.2	2.5
pH- (KCI)	6.24	6.07
Exchangeable acidity cmol/L	0.07	0.07
Total cations cmol/L	21.07	13.98
Acid saturation (%)	0	1
Soil Organic Carbon (%)	1.5	1.3
N (%)	0.08	0.09
Clay (%)	20	19

P—phosphorus; K—potassium; Ca—calcium; Mg—magnesium; Zn—zinc; Mn—manganese, Cu—copper; pH—potential hydrogen analyzed using potassium chloride (KCI); N—nitrogen.

## Data Availability

Data available upon request from the first author.
